# TRP75-mediated STAT3 activation promotes anti-apoptotic signaling and *Ehrlichia chaffeensis* infection

**DOI:** 10.1128/iai.00459-25

**Published:** 2025-10-20

**Authors:** Nicholas A. Pittner, Jaclyn R. McCoy, Duc-Cuong Bui, Jere W. McBride

**Affiliations:** 1 Department of Pathology, University of Texas Medical Branch 198642, Galveston, Texas, USA; 2 Department of Microbiology and Immunology, University of Texas Medical Branch 547647, Galveston, Texas, USA; 3 Center for Biodefense and Emerging Infectious Diseases, University of Texas Medical Branch, Galveston, Texas, USA; 4 Sealy Institute for Vaccine Sciences, University of Texas Medical Branch 559814, Galveston, Texas, USA; 5 Institute for Human Infections and Immunity, University of Texas Medical Branch 12338, Galveston, Texas, USA; University of California Davis, Davis, California, USA

**Keywords:** *Ehrlichia chaffeensis*, effector protein, apoptosis, STAT3, MCL-1, innate immunity

## Abstract

*Ehrlichia chaffeensis* is an obligately intracellular bacterium that manipulates mononuclear phagocytes by hijacking host cell signaling pathways to promote infection. Previous studies from our laboratory have shown that multiple signal transducer and activator of transcription (STAT) family members interact with *E. chaffeensis* effector proteins. However, the functional role of STATs during infection remains poorly understood. Notably, STAT3, a highly immunomodulatory and pro-survival factor, interacts with the *E. chaffeensis* effector protein TRP75. In this study, we examined activation of STAT family members and transcription of STAT target genes during *E. chaffeensis* infection. We observed significant activation of multiple STATs (STAT1, STAT3, STAT5, and STAT6), with STAT3 showing the highest level of activation. Therefore, we further investigated STAT3 activation dynamics and effects of its inhibition on infection. STAT3 phosphorylation and nuclear translocation were detected beginning 48 h post-infection, coinciding with upregulation of STAT3 target genes, including the anti-apoptotic gene *MCL-1*. Pharmacological inhibition of STAT3 significantly reduced *MCL-1* expression and increased caspase cleavage, implicating STAT3 as a regulator of anti-apoptotic signaling during infection. Furthermore, both pharmacological inhibition and genetic knockout of STAT3 significantly reduced bacterial load, highlighting its critical role in supporting infection. Ectopic expression of TRP75 in human embryonic kidney 293 cells induced STAT3 phosphorylation, demonstrating a specific role for TRP75 in STAT3 activation. Collectively, these findings support a model in which *E. chaffeensis* exploits STAT3 via the TRP75 effector to activate an anti-apoptotic program and other cellular pathways that promote infection.

## INTRODUCTION

*Ehrlichia chaffeensis* is an obligately intracellular, gram-negative bacterial pathogen and the etiologic agent of the emerging, life-threatening zoonosis, human monocytic ehrlichiosis (HME) (1). *E. chaffeensis* employs an arsenal of secreted effector proteins involved in a cellular reprogramming strategy required to evade innate defenses of the mononuclear phagocyte. Among these, the tandem repeat proteins (TRPs) are a group of intrinsically disordered, highly interactive effectors named for their unique tandem repeat sequences (2). TRPs are secreted via the type 1 secretion system, decorating the surface of *E. chaffeensis,* and positioned to interact with cellular surface receptors to initiate an infection program (3). After ehrlichial internalization, TRPs are secreted where they interact with a diverse array of host proteins in the cytoplasm and nucleus (4–7). Most notably, TRP effectors play critical roles during *E. chaffeensis* infection by activating and regulating host cell signaling pathways to establish an anti-apoptotic profile (2, 8, 9).

*E. chaffeensis* has four TRP effectors (TRP120, TRP75, TRP47, and TRP32), with the most studied being the multifunctional TRP120 (2). Although the importance of TRP120 in promoting infection has been well-defined, other TRP effectors remain comparatively understudied. The *E. chaffeensis* TRP75 effector is particularly notable as the largest (predicted) TRP member, with 11 (18 amino acid) tandem repeat sequences that are associated with increased tandem repeat domain mass due to a predominant basic amino acid composition. TRP75 is also tyrosine phosphorylated during infection and is predicted to be a lipoprotein (10, 11). Similar to other TRPs, TRP75 interacts with a wide variety of host proteins, though the functional consequences of these interactions are not fully understood (4–7). TRP75-binding partners are associated with cytoskeleton organization, metabolism, post-translational modification, homeostasis, and apoptosis (6). Interestingly, TRP75 is the only TRP known to interact with signal transducer and activator of transcription 3 (STAT3), an immunomodulatory transcription factor with well-established roles in both malignancy and infection (6, 12–15).

STAT3 is one of seven members of the STAT family and is known to broadly regulate immunity, inflammation, proliferation, and differentiation. STAT3 activation is typically initiated by ligands including interleukin (IL)-6, IL-10, and colony-stimulating factor 1 (CSF1) to promote immunosuppression and cell growth (16–18). Canonically, these signals activate Janus kinases (JAKs), which phosphorylate STAT3 at Y705; however, other kinases, like Src family kinases, can also mediate activation. Before phosphorylation, STAT3 exists as a cytoplasmic monomer, but phosphorylation enables dimerization via interactions between phospho-tyrosine residues and STAT3 SH2 domains. STAT3 dimerization exposes a nuclear localization sequence, which facilitates nuclear translocation and subsequent expression of STAT3 target genes (12, 19, 20). Generally, these target genes represent cancer hallmarks, such as immunosuppressive cytokines (*IL10*, *IL23*, and *TGFβ*), metabolic regulators (*SLC2A1, SREBP*), cell cycle regulators (*CCND1*, *MYC*), and anti-apoptotic proteins (*BCL2*, *BCL2L1*, and *MCL-1*) (20–28). Thus, it is not unexpected that aberrant STAT3 signaling is frequently implicated in malignancy and co-opted by intracellular pathogens to promote host cell survival and immune evasion (12–15).

In this study, we demonstrate that TRP75-mediated activation of STAT3 plays a critical role in *E. chaffeensis* infection. Although we determined that STAT3 contributes to the anti-apoptotic profile established during infection, our findings indicate that STAT3 likely possesses other functions associated with its role in promoting infection. These findings highlight *E. chaffeensis* exploitation of STAT3 as part of an overall strategy to repurpose host cell transcription to promote infection by modulating multiple host cell processes.

## RESULTS

### STAT family members are differentially activated during *E. chaffeensis* infection

**Fig 1:**
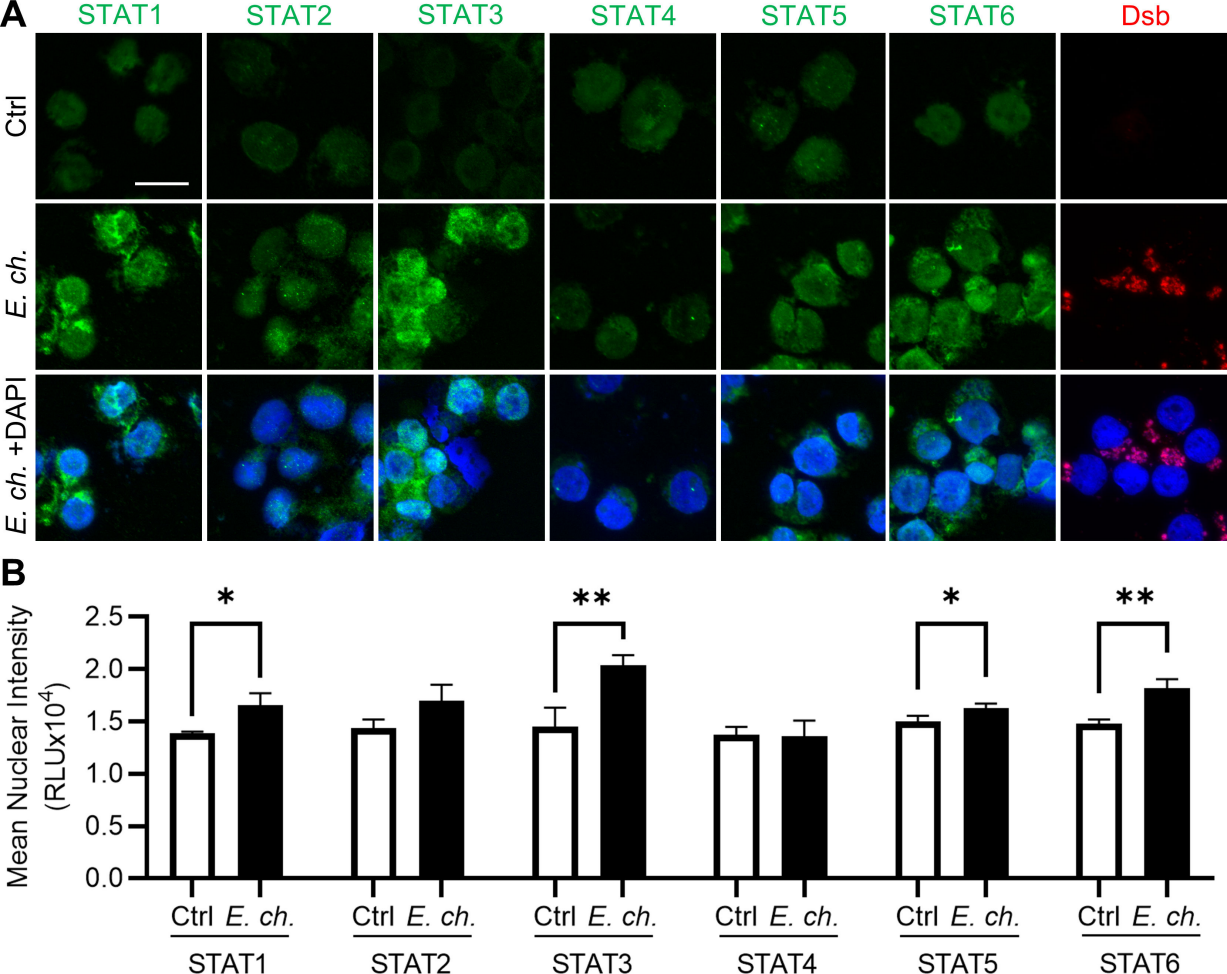
Nuclear translocation of STAT family members during *E. chaffeensis* infection. (**A**) Uninfected or *E. chaffeensis*-infected THP-1 cells at 72 hpi (MOI 10) were probed for STAT1–6 (green) and *E. chaffeensis* marker Dsb (red) by immunofluorescent microscopy. 4´,6-Diamidino-2-phenylindole (DAPI) (blue) was used to stain nuclei. Scale bar = 20 µm. (**B**) Nuclear regions of interest (ROIs) were defined by DAPI signal, and mean nuclear STAT3 intensity per cell was quantified using ImageJ. Statistical significance was determined using two-tailed Student’s *t*-test; *P < 0.05, **P < 0.01. Data represent the mean ± SD of three independent biological replicates (*n* = 3), and representative images are shown.

To identify STAT family members activated during *E. chaffeensis* infection, THP-1 cells were incubated with cell-free *E. chaffeensis* at a multiplicity of infection (MOI) of 10 and harvested 72 h post-infection (hpi) for subsequent immunofluorescent analysis. Using nuclear translocation as an indicator of STAT activation, we observed significant activation of STAT1, STAT3, STAT5, and STAT6 in *Ehrlichia*-infected cells compared to uninfected controls (Fig. 1). While four of the six STAT members evaluated were significantly activated, STAT3 and STAT6 exhibited the highest level of nuclear translocation. Although STAT2 nuclear translocation increased during infection, the level was not significant. Given the established role of STAT3 in cell survival and immunomodulation, as well as its well-defined interaction with *E. chaffeensis* effector TRP75, STAT3 was investigated further.

### *Ehrlichia chaffeensis* promotes STAT3 activation

**Fig 2:**
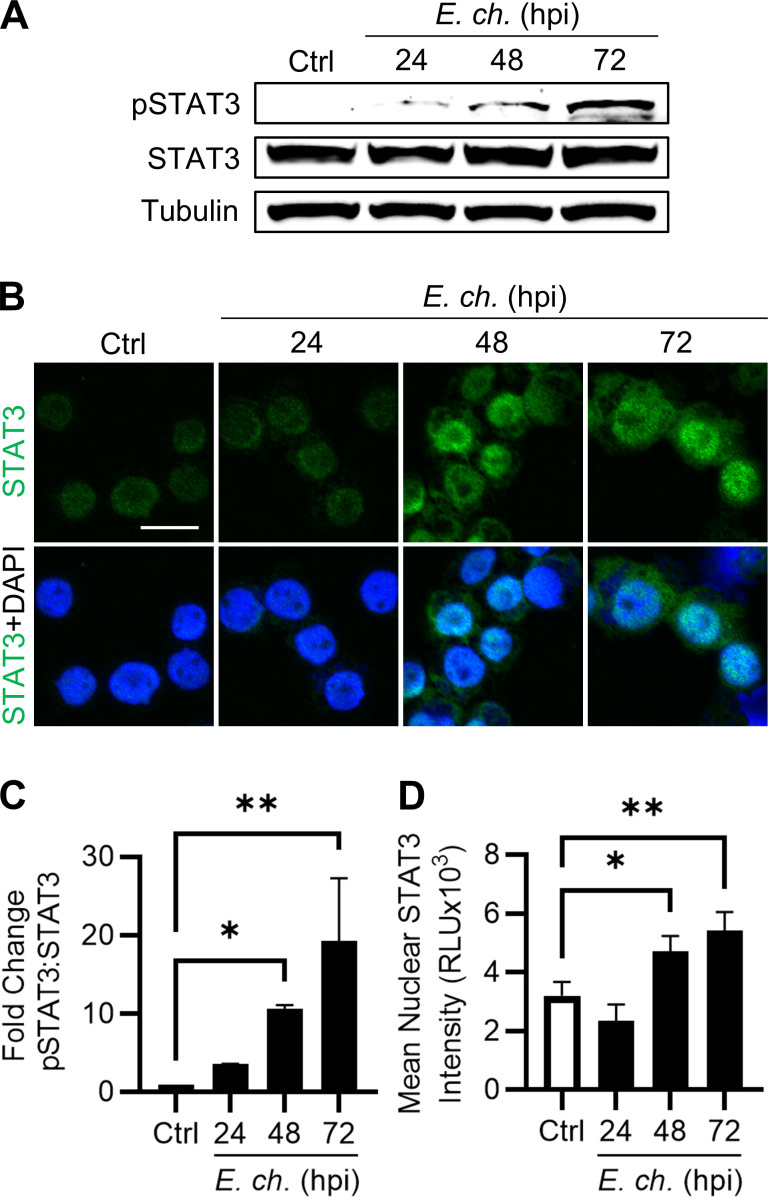
*E. chaffeensis* promotes STAT3 phosphorylation and nuclear translocation in host cells. (**A**) Immunoblot analysis of phosphorylated STAT3 (Y705) and total STAT3 in THP-1 cells infected with *E. chaffeensis* (MOI 10) at 0, 24, 48, and 72 hpi. (**B**) Immunofluorescent microscopy images of STAT3 (green) in infected cells at indicated time points. Scale bar = 20 µm. (**C**) Fold change in pSTAT3:STAT3 normalized to α-tubulin. (**D**) Mean nuclear STAT3 intensity per cell quantified using ImageJ. Nuclear regions of interest were defined by 4′,6-diamidino-2-phenylindole (DAPI) staining. Statistical significance was determined using one-way analysis of variance followed by Dunnett’s *post hoc* test to compare each time point to 0 hpi; *P < 0.05, **P < 0.01. Data represent the mean ± SD of three independent biological replicates (*n* = 3), and representative images are shown.

Regardless of the activating stimuli, STAT3 phosphorylation leading to activation results in dimerization and nuclear translocation. Thus, to evaluate STAT3 involvement throughout infection, we measured both STAT3 phosphorylation at Y705 and nuclear translocation by western blot and immunofluorescent microscopy, respectively (Fig. 2A and B). Briefly, THP-1 cells were incubated with cell-free *E. chaffeensis* (MOI 10) and harvested at 0, 24, 48, and 72 hpi to assess STAT3 activation throughout a full cellular infection cycle at early, mid, and late stages, respectively. While total levels of STAT3 remained consistent throughout the course of infection, activated STAT3 (phosphorylated at Y705) significantly increased at the 48 and 72 hpi time points (Fig. 2A and C). Additionally, levels of nuclear STAT3 were significantly elevated at 48 and 72 hpi in *Ehrlichia*-infected cells compared to uninfected control cells (Fig. 2B and D), demonstrating STAT3 activation during *E. chaffeensis* infection.

### STAT3 target genes are upregulated during *E. chaffeensis* infection

**Fig 3:**
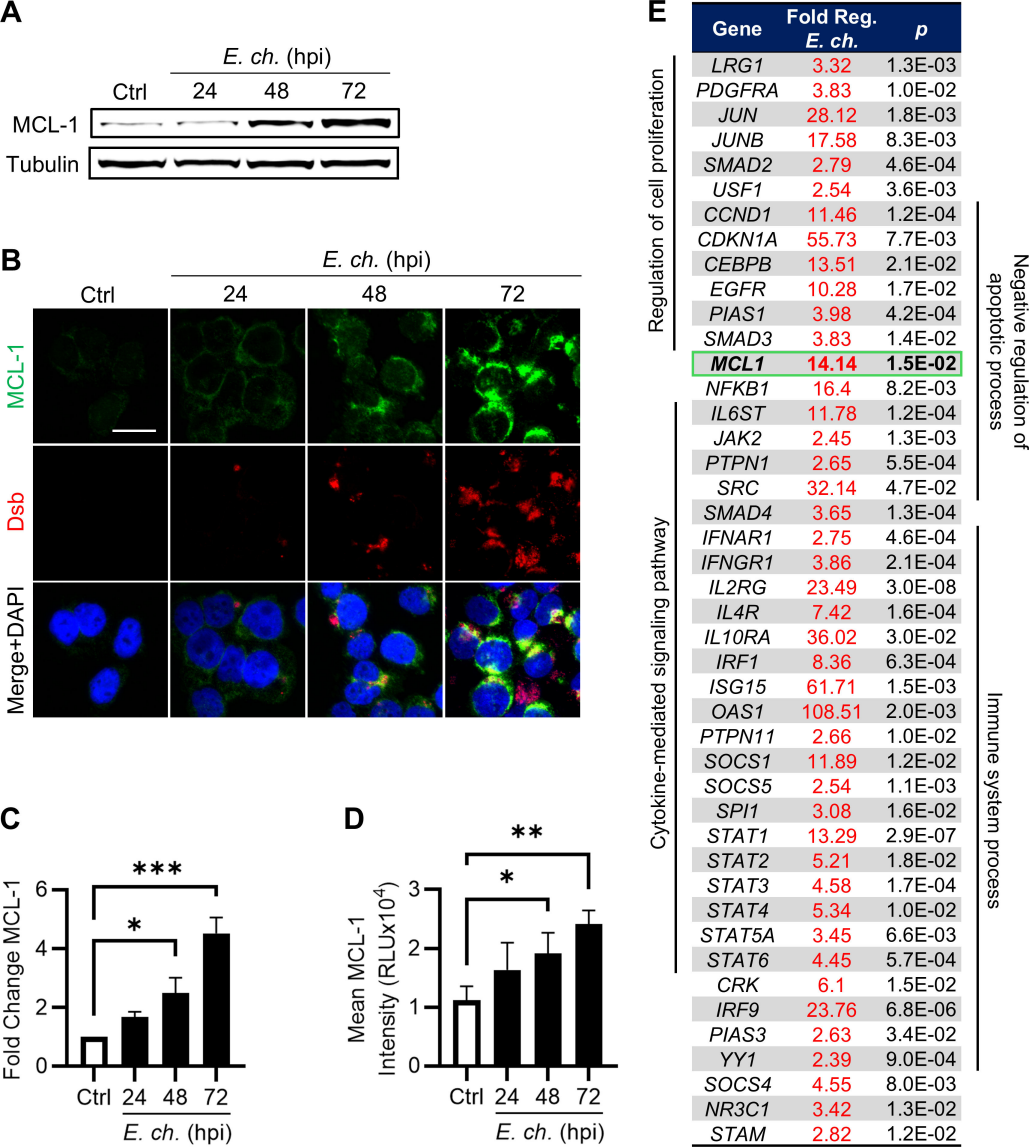
Transcriptional and post-transcriptional analysis of STAT3 target MCL-1 during *E. chaffeensis* infection. (**A**) Immunoblot analysis of MCL-1 protein levels in *E. chaffeensis*-infected THP-1 cells (MOI 10) at 0, 24, 48, and 72 hpi. (**B**) Fold change in MCL-1 normalized to α-tubulin. (**C**) Immunofluorescent microscopy analysis of MCL-1 (green) and Dsb (red) in infected cells at indicated time points. Scale bar = 20 µm. (**D**) Mean MCL-1 intensity per cell quantified using ImageJ. (**E**) Table of JAK/STAT target genes significantly upregulated at least twofold in *E. chaffeensis*-infected cells at 72 hpi compared to uninfected cells. Statistical significance was determined using one-way analysis of variance followed by Dunnett’s *post hoc* test to determine whether any time point differed from 0 hpi; *P < 0.05, **P < 0.01, ***P < 0.001. Data represent the mean ± SD of three independent biological replicates (*n* = 3), and representative images are shown.

As a negative regulator of apoptosis, we hypothesized that STAT3 contributes to apoptotic inhibition at late time points during infection. Given that *E. chaffeensis* is known to increase the expression of anti-apoptotic B-cell lymphoma 2 (BCL-2) family members and myeloid cell leukemia 1 (*MCL-1*) is a transcriptional target of STAT3, we investigated MCL-1 expression during infection (2, 27). Indeed, both western blot and immunofluorescent microscopy demonstrated significant increases in MCL-1 protein concomitant with STAT3 activation at 48 and 72 hpi (Fig. 3A through D).

To obtain a more comprehensive understanding of JAK/STAT signaling during *Ehrlichia* infection, STAT family target gene expression was examined. Since STAT3 is activated, we expected to detect differential expression of STAT3 target genes; however, other STAT proteins activated by *E. chaffeensis*, such as STAT5 and STAT6, also mediate inflammatory and oncogenic signaling and may be relevant during infection (16, 19, 29). Therefore, we performed a JAK/STAT signaling reverse transcriptase quantitative PCR (RT-qPCR) array to assess broad modulation of STAT family target genes during infection. Since STAT3 activation was detected at 48 and 72 hpi, transcriptional profiling focused on the 72 hpi time point to capture maximal changes in gene expression. Normalized expression of genes significantly upregulated by at least twofold in infected THP-1 cells compared to uninfected cells at 72 hpi is depicted in Fig. 3E. Of the 84 genes included on the array, 44 were significantly upregulated. These upregulated genes were associated with biological processes such as metabolism, cytokine signaling, immune response, cellular proliferation, and negative regulation of apoptosis. Notably, *MCL-1* was upregulated more than 14-fold in infected cells. Collectively, these results demonstrate that *E. chaffeensis* modulates expression of STAT target genes, including *MCL-1*, during infection.

### STAT3 inhibition abrogates MCL-1 upregulation

To determine which of the differentially expressed STAT target genes are specifically regulated by STAT3, JAK/STAT gene transcription was examined in response to the selective STAT3 inhibitor, C188-9. C188-9 abrogates STAT3 dimerization and subsequent nuclear translocation by occupying the phospho-tyrosine binding site in the STAT3 SH2 domain (30, 31). By comparing the expression of STAT target genes in infected cells with and without C188-9, a subset of genes whose differential expression depended on STAT3 activity was identified. This comparison allowed the identification of genes specifically influenced by STAT3 from that of other STAT family members.

**Fig 4:**
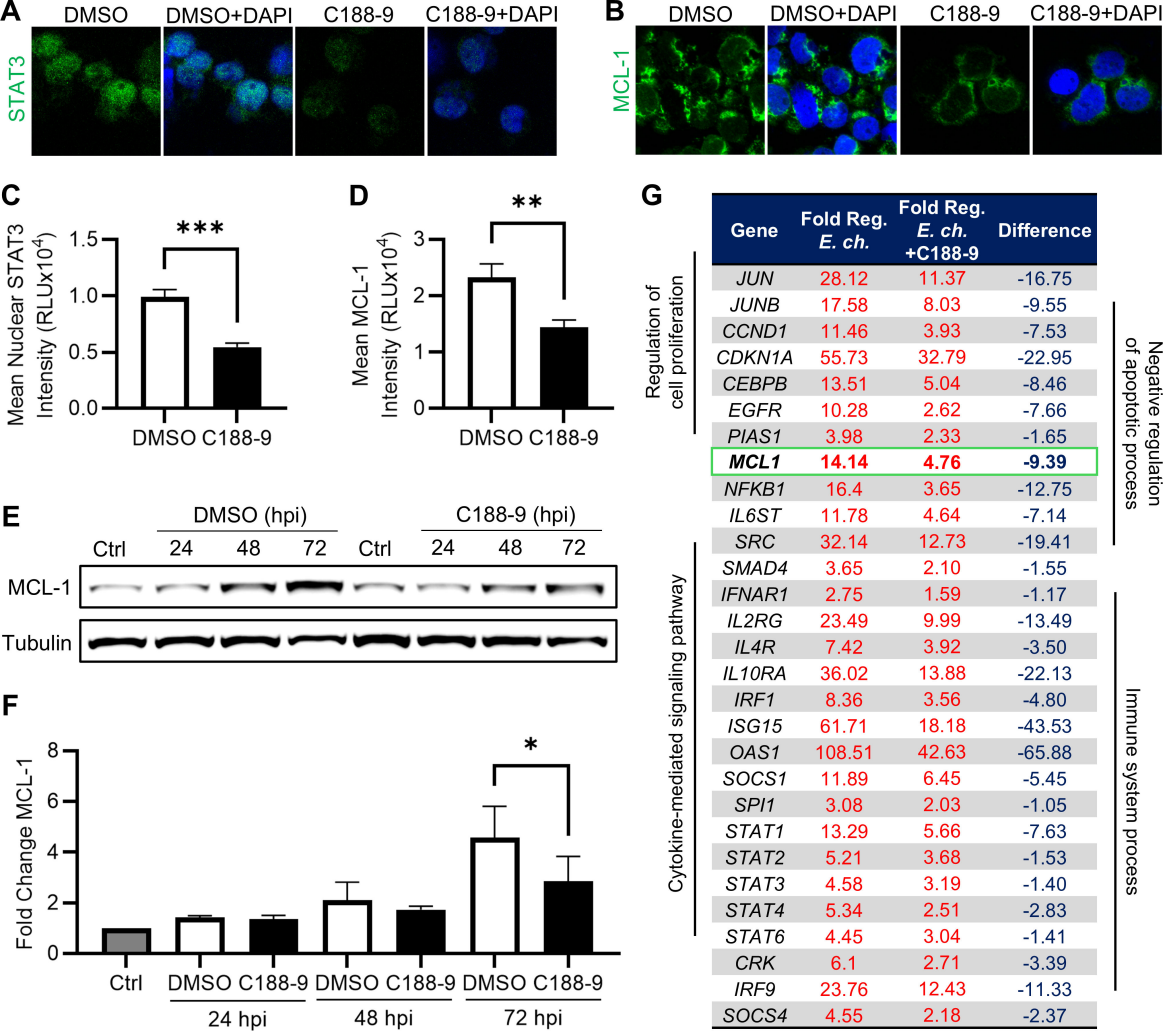
STAT3 inhibitor C188-9 abrogates *Ehrlichia*-induced STAT3 nuclear translocation and target gene expression. (**A**) Confocal microscopy images of nuclear STAT3 (green) and (**B**) MCL-1 (green) in *E. chaffeensis*-infected THP-1 cells (MOI 10) at 72 hpi following 3 h pretreatment with either DMSO (0.1%) or C188-9 (10 µM). Scale bar = 20 µm. (**C**) Mean nuclear STAT3 and (**D**) mean whole-cell MCL-1 intensity quantified using ImageJ. (**E**) Immunoblot analysis of MCL-1 in infected cells at indicated time points following 3 h pretreatment with either DMSO or C188-9. (**F**) Fold change in MCL-1 protein normalized to α-tubulin. (**G**) Table of JAK/STAT target genes exhibiting at least a onefold reduction in expression in *E. chaffeensis*-infected cells at 72 hpi following C188-9 pretreatment. Statistical significance was determined using two-tailed Student’s *t*-test and one-way analysis of variance followed by Dunnett’s *post hoc* test comparing each time point to 0 hpi; *P < 0.05, **P < 0.01. Data represent the mean ± SD of three independent biological replicates (*n* = 3), and representative images are shown.

THP-1 cells pretreated with C188-9 (10 µM) or dimethyl sulfoxide (DMSO) (0.1%) 3 h prior to infection with *E. chaffeensis* exhibited significantly less nuclear STAT3 than DMSO-treated cells at 72 hpi by immunofluorescent microscopy (Fig. 4A and C). Similarly, immunofluorescent microscopy and western blot revealed a significant difference in MCL-1 between C188-9- and DMSO-treated infected cells at 72 hpi, suggesting that STAT3 was predominantly responsible for MCL-1 upregulation during late infection (Fig. 4B and D through F). Having established the efficacy of C188-9 in preventing STAT3 nuclear translocation and MCL-1 upregulation, the JAK/STAT signaling RT-qPCR array was repeated using infected cells pretreated with C188-9. Of the 44 genes upregulated during infection at 72 hpi, 29 were expressed at least onefold less in response to STAT3 inhibition, suggesting that STAT3 was responsible for the majority of transcriptional regulation mediated by the STAT family during infection (Fig. 4G). This subset included genes from the previously identified biological processes, as well as *MCL-1*, which had a >9-fold reduction in expression in infected cells pretreated with C188-9 compared to DMSO. Collectively, these results illustrate that *E. chaffeensis* activation of STAT3 results in differential expression of STAT3 target genes including MCL-1.

### STAT3 inhibition reduces *E. chaffeensis* infection

**Fig 5:**
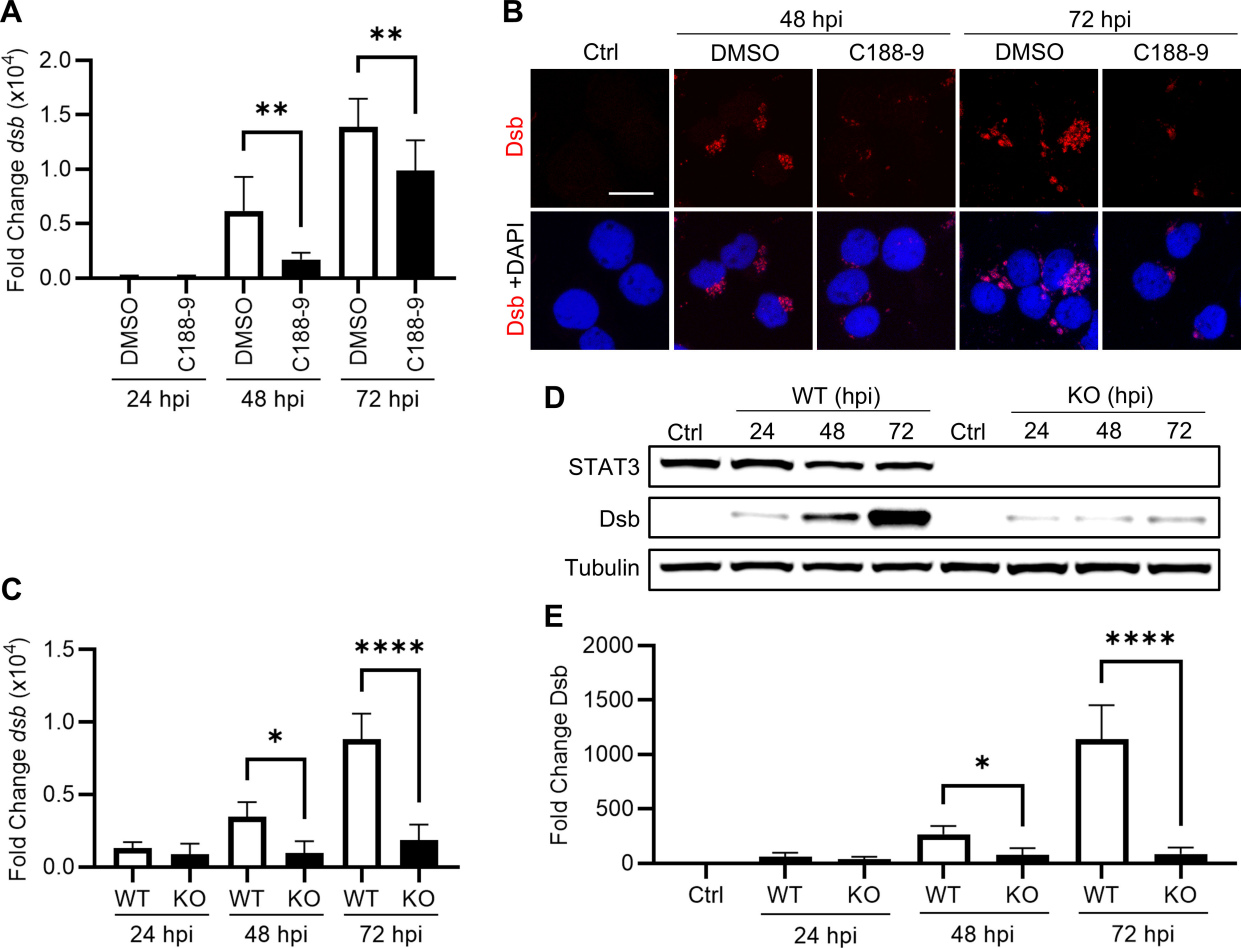
STAT3 inhibition and knockout (KO) reduce ehrlichial infection. (**A**) Ehrlichial infection in THP-1 cells (MOI 10) quantified by qPCR amplification of the *dsb* gene normalized to *GAPDH* at 24, 48, and 72 hpi following pretreatment with DMSO (0.1%) or C188-9 (10 µM). Data are shown as fold change relative to 0 hpi. (**B**) Confocal microscopy images of Dsb (red) in *E. chaffeensis*-infected THP-1 cells at 48 and 72 hpi following 3 h pretreatment with either DMSO or C188-9. Scale bar = 20 µm. (**C**) Ehrlichial infection in wild-type (WT) and STAT3 KO HeLa cells (MOI 10) quantified by qPCR amplification of the *dsb* gene normalized to *GAPDH* at 24, 48, and 72 hpi. (**D**) Immunoblot analysis of Dsb WT and KO HeLa cells (MOI 10) at indicated time points. (**E**) Fold change in Dsb normalized to α-tubulin. Statistical significance was determined using one-way analysis of variance followed by Dunnett’s *post hoc* test to compare DMSO vs C1889 or WT vs KO at each time point; *P < 0.05, **P < 0.01, ***P < 0.001, ****P < 0.0001. Data represent the mean ± SD of three independent biological replicates (*n* = 3), and representative images are shown.

To determine whether STAT3 meaningfully contributes to bacterial replication or survival in host cells, we assessed infection using RT-qPCR following pretreatment with DMSO or C188-9. There was a significant reduction in ehrlichial infection at 48 and 72 hpi in *E. chaffeensis*-infected cells pretreated with C188-9 (Fig. 5A). Similarly, a reduction in infection was observed by immunofluorescent microscopy (Fig. 5B). Together, these data suggest that STAT3 plays a critical role in *E. chaffeensis* replication or survival in host cells, particularly later in infection.

To account for off-target effects of C188-9, we also evaluated the effects on *E. chaffeensis* infection using available STAT3 knockout (KO) HeLa cells (Ubigene) to validate our findings in a STAT3-deficient genetic background. We observed significant reductions in ehrlichial infection using RT-qPCR and western blot at 48 and 72 hpi, confirming the results obtained with the inhibitor (Fig. 5C through E). In fact, decreases in bacterial infection in STAT3 KO cells were greater than those measured in cells treated with C188-9. These results confirmed the functional importance of STAT3 in *E. chaffeensis* infection and demonstrated that STAT3 abrogation inhibits infection.

### STAT3 inhibition and KO increase executioner caspase cleavage

**Fig 6:**
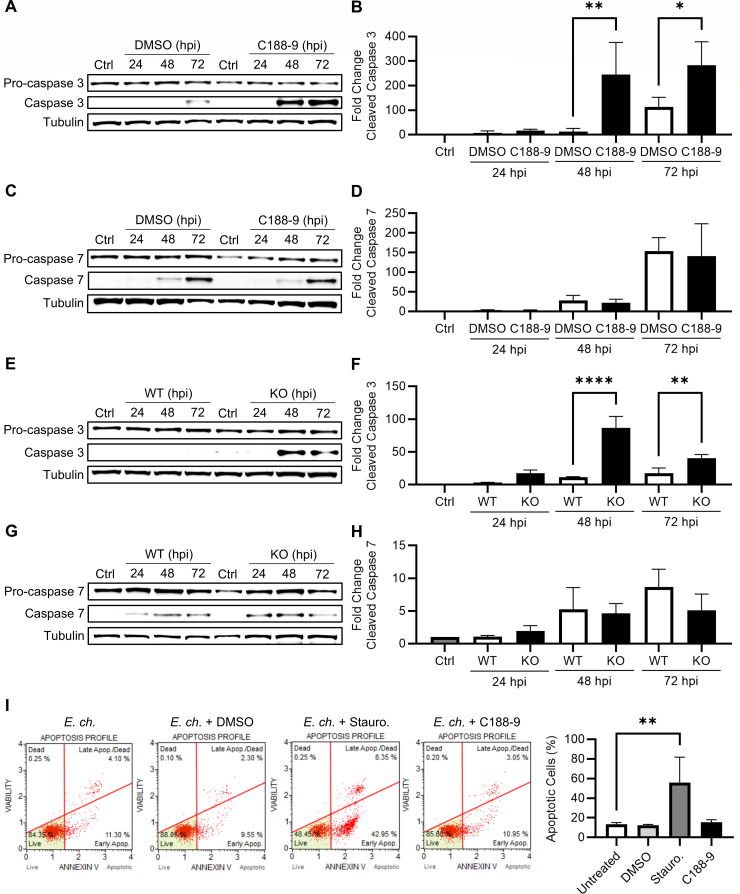
STAT3 inhibition and KO increase caspase cleavage without inducing apoptosis in *Ehrlichia*-infected cells. THP-1 cells were pretreated with DMSO (0.1%) or C188-9 (µM), and WT or STAT3 KO HeLa cells were infected with *E. chaffeensis* (MOI 10) prior to harvest at 0, 24, 48, and 72 hpi. (**A, E**) Immunoblot analysis of caspase 3 in *E. chaffeensis*-infected THP-1 and HeLa cells, respectively. (**B, F**) Fold change in cleaved caspase 3 normalized to α-tubulin. (**C, G**) Immunoblot analysis of caspase 7 in *E. chaffeensis*-infected THP-1 and HeLa cells, respectively. (**D, H**) Fold change in cleaved caspase 7 normalized to α-tubulin. (**I**) Percentage of apoptotic THP-1 cells pretreated with DMSO, C188-9, or staurosporine (0.1%, 10 µM, 100 nM) at 48 hpi (MOI 10) determined using the Annexin V and Dead Cell Kit. Statistical significance was assessed using one-way analysis of variance followed by Dunnett’s *post hoc* test to compare groups at each time point; *P < 0.05, **P < 0.01, ***P < 0.001, ****P < 0.0001. Data represent the mean ± SD of three independent biological replicates (*n* = 3), and representative images are shown.

Since STAT3 is responsible for MCL-1 upregulation at 72 hpi and is vital for bacterial infection, we evaluated whether STAT3 activation is an essential component of apoptotic inhibition by *E. chaffeensis*. Significantly greater levels of cleaved caspase 3 were observed by western blot at 48 and 72 hpi, in both C188-9-treated (Fig. 6A and B) and STAT3 KO cells (Fig. 6E and F). Conversely, there were no significant differences in caspase 7 cleavage in either THP-1 (Fig. 6C and D) or HeLa cells (Fig. 6G and H). Although elevated levels of cleaved caspase 3 suggest greater apoptotic pressure in the absence of STAT3 signaling, an associated increase in the percentage of apoptotic cells, as measured by annexin V flow cytometry, was not observed (Fig. 6I). This discrepancy may reflect partial activation of apoptotic machinery that was insufficient to trigger membrane changes detectable by annexin V. Although STAT3 abrogation increased caspase 3 activation, it did not induce apoptosis during infection. While unexpected, this finding suggests that the STAT3 contribution to apoptotic inhibition is secondary to other anti-apoptotic mechanisms known to be employed by *E. chaffeensis* (2, 8, 9). Furthermore, considering the impact of STAT3 abrogation on infection, the outcome of these experiments suggests that STAT3 has other pro-infection functions unrelated to inhibiting apoptosis.

### Ectopic expression of TRP75 activates STAT3

**Fig 7:**
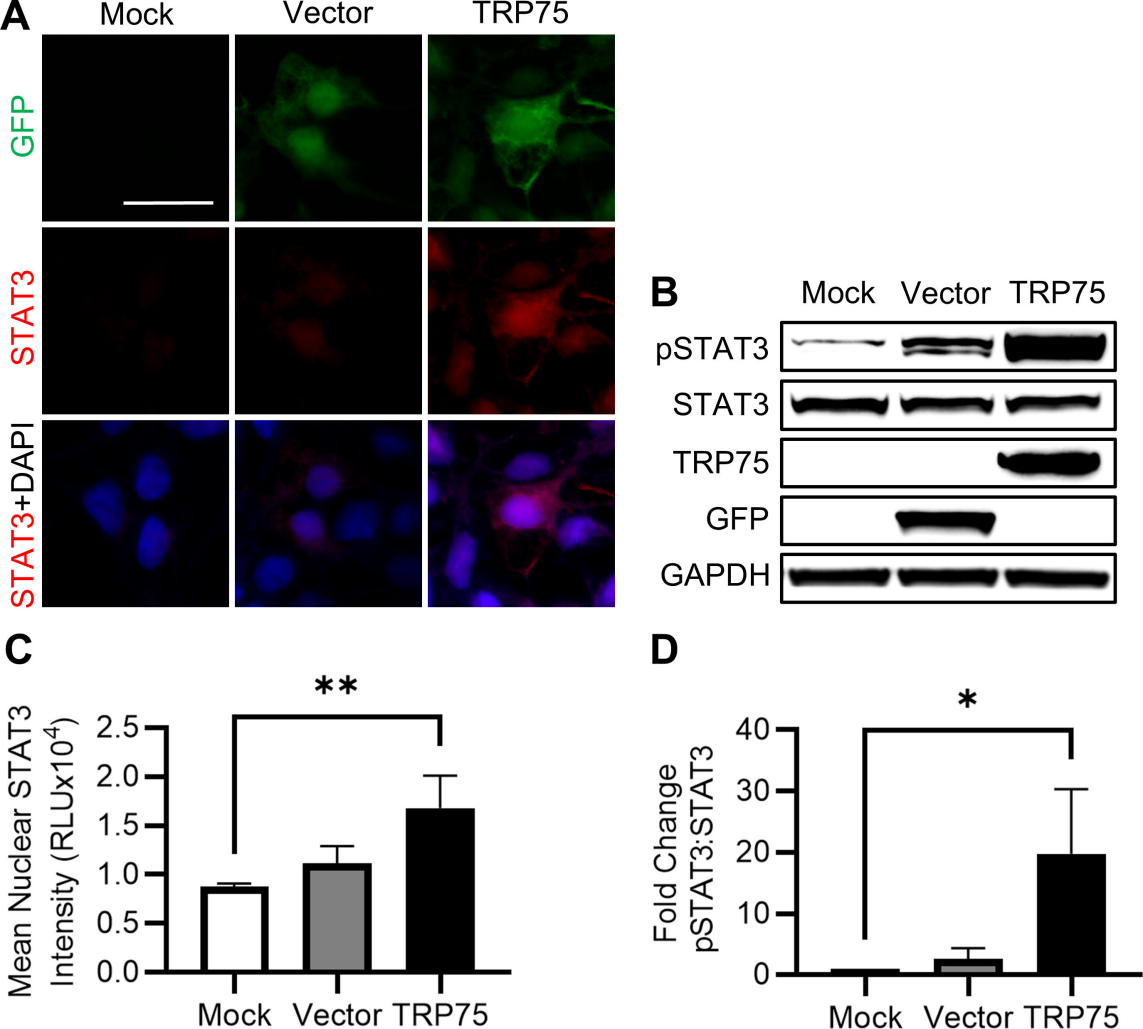
Ectopic expression of TRP75 promotes STAT3 phosphorylation and nuclear translocation. HEK-293 cells were transfected with a pAc-GFP plasmid encoding *E. chaffeensis* TRP75 using TransIt-LT1. Controls included an empty pAc-GFP vector and a mock transfection (transfection reagent alone). Cells were harvested at 30 h post-transfection. (**A**) Immunofluorescent microscopy images depicting GFP (green), indicating successful transfection, and STAT3 (red). Scale bar = 20 µm. (**B**) Immunoblot of phosphorylated STAT3 (Y705) and total STAT3. (**C**) Mean nuclear STAT3 intensity per cell quantified using ImageJ. (**D**) Fold change in pSTAT3:STAT3 ratio normalized to GAPDH. Statistical significance was determined using one-way analysis of variance followed by Dunnett’s *post hoc* test comparing vector and TRP75-transfected cells to mock; *P < 0.05, **P < 0.01. Data represent the mean ± SD of three independent biological replicates (*n* = 3), and representative images are shown.

Our laboratory previously reported a direct interaction between STAT3 and the *E. chaffeensis* effector TRP75 (6). Having established the importance of STAT3 for promoting *E. chaffeensis* infection, we sought to determine whether TRP75 is responsible for mediating STAT3 activation. To evaluate this, we ectopically expressed TRP75-GFP in human embryonic kidney 293 (HEK-293) cells and assessed downstream STAT3 activation. Intriguingly, cells expressing TRP75-GFP exhibited significantly greater levels of nuclear STAT3 by immunofluorescent microscopy at 30 h post-transfection (Fig. 7A). Similarly, TRP75-transfected cells possessed significantly greater levels of phosphorylated STAT3 compared to mock- and vector-transfected cells, while levels of total STAT3 remained consistent in all groups (Fig. 7B). Collectively, these results demonstrated that TRP75 plays a key role in STAT3 phosphorylation in uninfected cells.

### *E. chaffeensis* activates STAT3 and in primary human monocytes (PHMs) *ex vivo*

**Fig 8:**
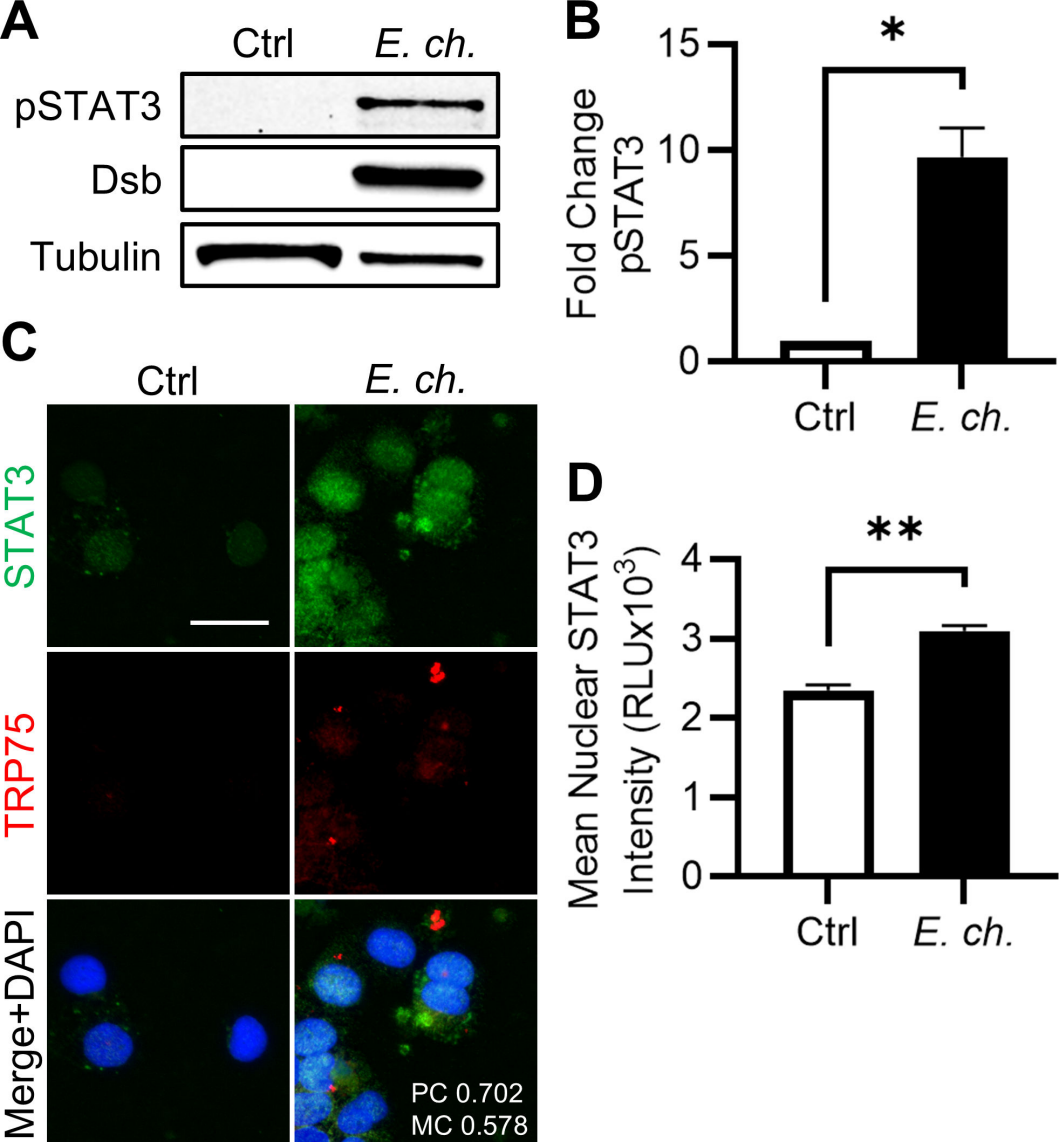
STAT3 is activated in *Ehrlichia*-infected primary human monocytes *ex vivo*. Primary human monocytes were infected with *E. chaffeensis* (MOI 10) and harvested at 48 hpi. (**A**) Immunoblot analysis of phosphorylated STAT3 (Y705). (**B**) Fold change in pSTAT3 levels normalized to α-tubulin. (**C**) Immunofluorescent microscopy images of STAT3 (green) and TRP75 (red) in infected monocytes. Scale bar = 20 µm. (**D**) Mean nuclear STAT3 intensity per cell quantified using ImageJ. Statistical significance was determined using two-tailed Student’s *t*-test; *P < 0.05, **P < 0.01. Data represent the mean ± SD of two independent biological replicates (*n* = 2), and representative images are shown.

To validate that STAT3 activation is consistent in a physiologically relevant model, an *ex vivo* analysis in PHMs was performed. Indeed, PHMs infected with *E. chaffeensis* exhibited significantly greater levels of phosphorylated STAT3 at 48 hpi (Fig. 8A and C). Similarly, greater levels of nuclear STAT3 were observed by immunofluorescent microscopy in infected PHMs (Fig. 8B through D). Collectively, these results confirm STAT3 activation is a conserved feature of *E. chaffeensis* infection in established cell lines as well as *ex vivo* in PHMs, reinforcing its biological relevance.

## DISCUSSION

The JAK-STAT pathway is an evolutionarily conserved signaling axis involved in essential cellular processes including immunity, metabolism, and survival. While all seven STAT transcription factors share a common activation mechanism, they induce distinct, even opposite, transcriptional programs (19, 32, 33). For example, STAT1 is an arbiter of interferon signaling, playing a critical role in antiviral defense and tumor suppression. In contrast, STAT3 is immunomodulatory, often implicated in immune suppression and driving oncogenesis. The disparity in mechanistic impact is important to recognize, as a historical tendency to refer broadly to “JAK-STAT signaling” can obscure these functional distinctions (33). Relevant to this study, *E. chaffeensis* has been previously reported to inhibit JAK-STAT signaling early in infection; however, in contrast, we reveal that multiple STAT family members are activated later in infection (34). Thus, the current study enhances our understanding of how *E. chaffeensis* precisely and temporally manipulates STAT signaling to reflect the diversity of function associated with STAT transcription factors on cellular processes during infection.

Our prior work has identified multiple interactions between STAT proteins and *E. chaffeensis* effectors, including one between STAT3 and TRP75 (5, 6). Interestingly, we also demonstrated that TRP75 is tyrosine phosphorylated and interacts with CSF1 receptor (CSF1R), a receptor tyrosine kinase known to initiate STAT3 activation (6, 10, 35). As a predicted lipoprotein, TRP75 may associate with the cell membrane, thereby promoting this interaction (11). The abundant evidence suggesting a relationship between TRP75, STAT3, and an associated receptor (CSF1R) prompted us to investigate the functional relevance of STAT3 and TRP75 during *E. chaffeensis* infection.

STAT3 is broadly implicated in human disease, the most notable being cancer. Dysregulation of STAT3 has been reported in as many as 70% of human malignancies and regulates multiple hallmarks of cancer (36). It promotes cell proliferation through genes such as *CCND1* and *MYC* and blocks apoptosis through upregulation of BCL-2 family proteins, including *MCL-1* (25–27). STAT3 also alters metabolism in cancer cells, having been linked with elevated glucose utilization via increased expression of glycolytic enzymes as well as dysregulated lipid biosynthesis through *SREBP1* and *ACC1* (23, 24, 37). Additionally, STAT3 contributes to immune evasion by upregulating *PDL1* (38). These examples are far from exhaustive but illustrate the diverse functions that have made STAT3 a compelling target for cancer therapy and, as our data suggest, a target co-opted by *E. chaffeensis*.

Multiple STAT family members were activated during *E. chaffeensis* infection, including STAT1, STAT3, STAT5, and STAT6; however, using a specific STAT3 inhibitor, most of STAT-related gene expression during infection was found to be associated with STAT3. We demonstrated that STAT3 phosphorylation and nuclear translocation were significantly elevated by *E. chaffeensis*, particularly in later phases of infection. Transcriptomic analysis revealed significant upregulation of JAK/STAT target genes during infection, most of which were attenuated by treatment with a specific STAT3 inhibitor, C188-9. The fact that STAT3 inhibition affected the expression of most upregulated JAK/STAT target genes suggests STAT3 plays a major role in STAT-related transcriptional control during infection. Genes linked to STAT3 during infection were associated with GO terms such as regulation of cell proliferation (*CCND1, JUNB*), negative regulation of apoptosis (*MCL-1, NFKB1*), cytokine-mediated signaling pathway (*IL10RA, SOCS1*), and immune system process (*CRK, IRF9*). Given our prior research establishing MCL-1 upregulation at the protein level during infection, we assessed the relevance of STAT3 in MCL-1 upregulation and apoptotic inhibition. A significant increase in MCL-1 was observed at 48 and 72 hpi, consistent with increased activation of STAT3. Furthermore, MCL-1 was significantly decreased by 72 hpi after STAT3 inhibition. This finding is consistent with reduced MCL-1 expression (ninefold; 72 hpi) following STAT3 inhibition. These results suggest MCL-1 may be stabilized initially at the protein level by previously established TRP120-mediated degradation of the negative regulator FBW7, but that upregulation of MCL-1 later in the infection cycle is related to transcriptional activation by STAT3 (39). Intriguingly, MCL-1 was also found to colocalize with *E. chaffeensis* morulae throughout infection, suggesting the possibility of a distinct, post-translational regulatory mechanism like that observed in *Chlamydia trachomatis* (40). Alternatively, this finding may reflect independent mitochondrial localization by both *E. chaffeensis* and MCL-1 (41, 42).

Pharmacologic inhibition of STAT3 significantly decreased infection at 48 and 72 hpi, which was also confirmed in STAT3 KO cells. Complete abrogation of STAT3 in KO cells prevented an increase in infection beyond that observed at 24 hpi, suggesting STAT3 is critical for ehrlichial infection at late phases. Additionally, both STAT3 inhibition and STAT3 KO cells exhibited increased caspase 3 cleavage at 48 and 72 hpi; however, an accompanying increase in the percentage of apoptotic cells was not detected. Although this result appears paradoxical, it is potentially explained by the broader anti-apoptotic phenotype induced by *E. chaffeensis*. Given the redundancy of anti-apoptotic strategies employed by *E. chaffeensis*, such as X-linked inhibitor of apoptosis (XIAP) stabilization and BCL-2 upregulation, the contribution of STAT3 to apoptotic inhibition may be secondary to other anti-apoptotic strategies (43–47). For example, although STAT3 modulates cleavage of caspase 3 during infection, the apoptotic pressure elicited by STAT3 inhibition may be insufficient to outweigh the effect of XIAP stabilization, which acts downstream of other anti-apoptotic mechanisms, such as BCL-2 family proteins (47).

The significant impact of STAT3 abrogation on infection in the absence of apoptosis indicates that STAT3 may contribute to infection in other ways. The principal role of STAT3 in *E. chaffeensis* infection is likely analogous to that observed in other intracellular infections. For example, *Salmonella typhimurium* and *Bartonella henselae* effectors promote STAT3 activation to stimulate M2 macrophage polarization (48, 49). Similarly, *Mycobacterium tuberculosis* activates STAT3 to suppress inflammatory cytokines and nitric oxide production through upregulation of *IL4*, *IL10*, and *ARG1* (50). Alternatively, STAT3 could play an important role in regulating host cell metabolism; influenza A virus activates STAT3 to increase expression of sterol regulatory element binding protein-2 (SREBP-2), thereby elevating host cholesterol biosynthesis to increase viral replication (51). Intriguingly, *E. chaffeensis* depends on host cholesterol for membrane integrity, and cholesterol depletion prevents *E. chaffeensis* from infecting host cells (52). STAT3 activation may play a role in *E. chaffeensis* infection by satisfying a crucial requirement for cholesterol. STAT3 signaling is evidently beneficial for a variety of intracellular pathogens by multiple mechanisms, and the significant effect of STAT3 inhibition on bacterial infection suggests *E. chaffeensis* is no exception.

Although a complete understanding of STAT3’s role in *E. chaffeensis* infection remains to be elucidated, we demonstrated the importance of STAT3 in *E. chaffeensis* infection and implicated the TRP75 effector in STAT3 activation. Ectopic expression of TRP75 alone was sufficient to induce both STAT3 phosphorylation and nuclear translocation in HEK-293 cells. Given that TRP75 interacts with STAT3 during infection and itself is tyrosine phosphorylated, TRP75 may serve as an adaptor which spatially associates STAT3 with host kinases to facilitate STAT3 phosphorylation in a mechanism like those reported in *S. typhimurium* and *B. henselae* (10, 48, 49). Alternatively, TRP75 could induce a signal cascade that culminates in STAT3 activation through a mechanism like that reported in *Helicobacter pylori* (14, 53). Indeed, we have previously shown that TRP75 interacts with the tyrosine kinase CSF1R, a class III receptor tyrosine kinase that plays a key role in the regulation of mononuclear phagocytes and initiates STAT3 activation (6, 35). It is possible that CSF1R is involved in TRP75 or STAT3 phosphorylation; however, future studies will be necessary to determine the kinase involved.

To validate the physiological relevance of our findings, we confirmed STAT3 activation in PHMs. We observed significant increases in phosphorylated and nuclear STAT3 in PHMs at 48 hpi. These results are consistent with those obtained in immortalized cell lines, supporting the notion that STAT3 activation is a biologically important feature of *E. chaffeensis* infection, emphasizing the translational relevance of addressing unanswered questions related to STAT3.

Our findings established that STAT3 is activated in a TRP75-dependent manner and plays a critical role in supporting *E. chaffeensis* survival. Future studies should explore cellular processes mediated by STAT3 that may be responsible for its impact on *E. chaffeensis* infection such as M2 polarization, immunosuppressive cytokine production, and cholesterol synthesis. Additionally, the mechanism of TRP75-mediated STAT3 activation should be fully elucidated. Finally, given the pronounced decrease in *E. chaffeensis* infection following STAT3 inhibition, it is tempting to consider STAT3 as a therapeutic target in HME. Preclinical *in vivo* studies will be essential to determine whether targeting STAT3 could reduce ehrlichial infection without exacerbating immunopathology.

**Fig 9:**
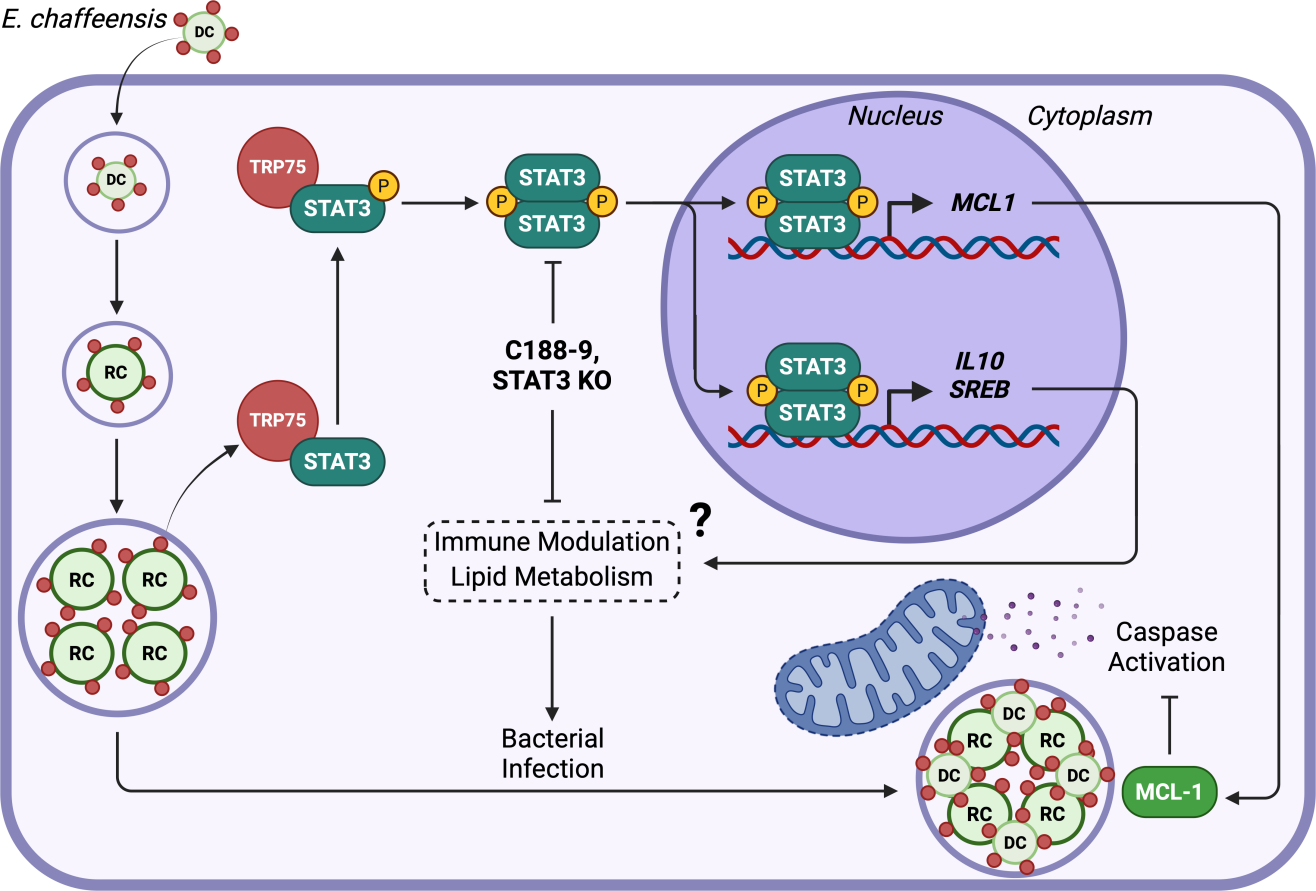
Proposed model of *E. chaffeensis* manipulation of STAT3. Following entry and initial replication in the host cell, TRP75 is secreted into the host cytosol, where it interacts with STAT3, facilitating its phosphorylation by an undefined mechanism potentially involving CSF1R. Phosphorylated STAT3 dimerizes and translocates to the nucleus, where it upregulates target genes, including MCL-1, that reinforce the anti-apoptotic phenotype induced by *E. chaffeensis*. However, other STAT3 target genes likely participate in a distinct cellular program critical for bacterial survival in host cells, such as immune modulation, lipid metabolism, or cell proliferation. STAT3 KO or pharmacologic inhibition abrogates this function and consequently reduces bacterial survival in host cells.

In conclusion, this investigation demonstrated that multiple STAT family members are activated during *E. chaffeensis* infection, including STAT3. We illustrated that the activation of STAT3 occurs during late infection and implicated the TRP75 effector in STAT3 phosphorylation. Furthermore, we established that STAT3 is a contributor to the anti-apoptotic profile induced by *E. chaffeensis* by modulating cleavage of caspase 3 but plays a distinct, critical role in bacterial survival (Fig. 9). Although the primary function of STAT3 in promoting *E. chaffeensis* infection remains unknown, our findings have identified STAT3 as a crucial component in *E. chaffeensis* pathobiology and linked its activation to an understudied effector protein. This study adds to a broader understanding of how intracellular pathogens with small genomes have evolved elegant strategies to co-opt host cell machinery to promote infection.

## MATERIALS AND METHODS

### Cell culture and *E. chaffeensis* infection

Human monocytic leukemia cells (THP-1; ATCC TIB-202) were maintained in RPMI 1640 with L-glutamine and 25 mM HEPES buffer, 1 mM sodium pyruvate, and 2.5 g/L d-(+)-glucose (30-2001; ATCC) supplemented with 10% fetal bovine serum (FBS; HyClone SH30910.03) at 37°C in a humidified 5% CO_2_ atmosphere. *Ehrlichia chaffeensis* (Arkansas strain) was cultivated in THP-1 cells, and cell-free ehrlichiae were isolated as previously described (46). For transfection experiments, human embryonic kidney cells (HEK-293; ATCC CRL-1573) were maintained in Dulbecco’s modified Eagle’s medium (DMEM; ATCC 30-2002) supplemented with 10% FBS. In experiments utilizing inhibitors or cell death inducers, chemicals were added to THP-1 cells 3 h prior to incubation with *E. chaffeensis*.

### Antibodies and reagents

The primary antibodies utilized in this research include monoclonal rabbit α-STAT1 (14994S; Cell Signaling Technology), monoclonal rabbit α-STAT2 (72604S; Cell Signaling Technology), rabbit α-STAT3 (12640S; Cell Signaling Technology), monoclonal rabbit α-STAT4 (2653S; Cell Signaling Technology), monoclonal rabbit α-STAT5 (94205S; Cell Signaling Technology), monoclonal rabbit α-STAT6 (5397S; Cell Signaling Technology), monoclonal rabbit α-phospho-STAT3 Y705 (9145S; Cell Signaling Technology), monoclonal rabbit α-MCL-1 (94296S; Cell Signaling Technology), monoclonal rabbit α-caspase 3 (9662S; Cell Signaling Technology), monoclonal mouse α-caspase 7 (9494S; Cell Signaling Technology), monoclonal mouse α-GAPDH (sc-47724; Santa Cruz Biotechnology), monoclonal mouse α-Tubulin (sc-5286; Santa Cruz Biotechnology), monoclonal mouse α-Vinculin (sc-73614; Santa Cruz Biotechnology), monoclonal mouse α-GFP (sc-9996; Santa Cruz Biotechnology), rabbit α-disulfide bond formation protein (Dsb) (54), and rabbit α-TRP75 (10).

Secondary antibodies included horseradish peroxidase-conjugated goat anti-rabbit IgG (5450-0010; SeraCare) and anti-mouse IgG (5220-0341; SeraCare), used for immunoblotting, and Alexa Fluor Plus 488- or 594-conjugated goat anti-rabbit or anti-mouse IgG (A32731, A32723, A32740, A32742; Invitrogen), used for immunofluorescence microscopy.

The pharmacologic reagents used in this study were C188-9 (10 µM; HY-112288; MedChemExpress) for STAT3 inhibition, staurosporine (100 nM; 9953S; Cell Signaling Technology) for cell death induction, and DMSO (Sigma-Aldrich D2650) at 0.1%, used as a vehicle.

### Immunofluorescent microscopy

THP-1 and PHM cells were infected as described previously and harvested at indicated time points, washed once in ice-cold phosphate buffered saline (PBS), and adhered to glass slides by cytocentrifugation (1,000 RPM for 5 min). HEK-293 cells were seeded in 24-well plates containing a coverslip and incubated for 24 h prior to transfection; coverslips were harvested 30 h post-transfection. Cells were fixed with 4% paraformaldehyde for 15 min at room temperature, followed by three washes in PBS. Fixed cells were blocked and permeabilized with 0.1% Triton X-100 in PBS for 5 min and washed with PBS before incubation with Image-iT FX Signal Enhancer (I36933; Invitrogen) for 30 min. Slides were then incubated with BlockAid Blocking Solution (B10710; Invitrogen) for 30 min prior to subsequent incubation with primary antibodies (1:200) diluted in BlockAid for 1 h. Slides were washed thrice with PBS in between primary antibody treatments. Cells were then incubated with secondary antibodies (1:200) diluted in BlockAid for 30 min prior to mounting with ProLong Glass Antifade with 4′,6-diamidino-2-phenylindole (P36980, Invitrogen). Immunofluorescent micrographs were obtained with the Olympus BX61 Upright fluorescent microscope and Zeiss LSM 880 laser confocal microscope and analyzed with ImageJ software. Randomized areas per slide were imaged and used for quantification.

### Immunoblot analysis

Cells were treated and infected as described above, harvested at 0, 24, 48, and 72 hpi, and washed with PBS. Cells were lysed with cell extraction buffer (FNN0011; Invitrogen) supplemented with Halt protease and phosphatase inhibitor cocktail (78440; Thermo Fisher) and phenylmethylsulfonyl fluoride (P7626; Sigma-Aldrich) for 30 min on ice, vortexing every 10 min. Lysates were cleared by centrifugation at 14,000 × *g* for 10 min at 4°C. Bicinchoninic acid assay determined protein concentrations of cleared lysates, and 25 µg of protein were boiled at 95°C for 5 min with NuPAGE LDS Sample buffer (NP0008; Invitrogen) and NuPAGE Sample Reducing Agent (NP0009; Invitrogen). Heated lysates were resolved by SDS-PAGE before transfer to a nitrocellulose membrane. Membranes were blocked for 1 h with 5% nonfat milk in Tris-buffered saline with 0.1% Tween 20 (TBST) before overnight exposure to primary antibodies at 4°C. Membranes were washed thrice in TBST for a total of 45 min, followed by 1 h of incubation with horseradish peroxidase (HRP)-conjugated secondary antibodies diluted 1:10,000 in 5% nonfat milk in TBST. Visualization was conducted with WesternBright ECL (K-12045-D50; Advansta), WesternBright Sirius (K-12043-D10; Advansta), and the ChemiDoc MP Imaging System (Bio-Rad), while densitometry was performed using ImageLab software (ver 6.1; Bio-Rad).

### RNA isolation, cDNA synthesis, and qPCR

Total RNA was isolated using the RNeasy Mini Kit (74106; QIAGEN), and on-column DNA digestion was performed using the RNase-free DNase kit (79254; QIAGEN) according to the manufacturer’s protocol. The concentration and purity of the isolated RNA were determined using a Nanodrop 100 spectrophotometer (Thermo Fisher). Synthesis of cDNA from total RNA (0.5 µg) was accomplished using the iScript cDNA Synthesis Kit (1708891, Bio-Rad) according to the manufacturer’s instructions. Bacterial load quantification via qPCR was performed using the Brilliant II SYBR Green QPCR master mix (600828; Agilent). PCR primer sequences were synthesized commercially by Integrated DNA Technologies and include *dsb* (F: 5′-GTTTCATTAGCCAAGAATTCCGACACT-3′; R: 5′-GCTGCTCCACCAATAAATGTATCCCT-3′) and *GAPDH* (F: 5′-ACCACAGTCCATGCCATCAC-3′; R: 5′-TCCACCACCCTGTTGCTGTA-3′). Relative gene expression was calculated by determining the cycle threshold value and normalizing to *GAPDH* as previously described (43).

### JAK/STAT signaling pathway PCR array

The human JAK/STAT signaling pathway PCR array (330231; QIAGEN) profiled the expression of 84 genes involved in JAK/STAT signaling, including receptors, ligands, and known target genes. PCR arrays were performed according to the manufacturer’s protocol. Real-time qPCR was performed using the RT2 Profiler PCR array in combination with Brilliant II SYBR Green QPCR master mix (600828; Agilent) on a CFX Opus real-time PCR system (Bio-Rad). PCR conditions and analysis were conducted as previously described (43).

### STAT3 KO cells

Readily available CRISPR-Cas9 STAT3 KO HeLa cells were obtained from a commercial source (YKO-H142; Ubigene). Wild-type and STAT3 KO cells were maintained in DMEM supplemented with 10% FBS. HeLa cells were infected with cell-free *E. chaffeensis* (MOI 10) and harvested at the indicated time points for western blot and microscopic analysis as discussed previously.

### Transfection

HEK-293 cells were seeded at 2.5 × 10^5^ cells/mL in 24-well plates and cultured in DMEM supplemented with 10% FBS for 24 h prior to transfection. Cells were transfected with either the pAc-GFP-TRP75 plasmid, encoding the *E. chaffeensis* TRP75 effector protein fused to GFP, or an empty pAc-GFP vector using TransIT-LT1 (MIR 2304, Mirus Bio) according to the manufacturer’s protocol. Briefly, 1 µg of plasmid DNA and 3 µL of transfection reagent were diluted in Opti-MEM and incubated for 30 min before being added dropwise to cells in complete medium. Cells were harvested for immunofluorescence and immunoblotting at 30 h post-transfection. Transfection efficiency was verified by GFP fluorescence, and TRP75 expression was confirmed by GFP signal and anti-TRP75 immunoblot. A mock transfection consisting of transfection reagent alone was also used as a negative control.

### Annexin V flow cytometry

THP-1 cells were infected as previously described following 3 h pretreatment with either DMSO, C188-9, or staurosporine and evaluated for apoptotic cell populations at 48 hpi. The Guava Muse Cell Analyzer was used in conjunction with the Annexin V and Dead Cell Kit (MCH100105, Cytek) to quantify live, dead, early, and late apoptotic cell populations. The percentages of live, early apoptotic, late apoptotic, total apoptotic, and dead cells were determined.

### Human peripheral blood mononuclear cells and primary human monocytes

Primary human monocytes were isolated from de-identified donor human blood obtained from the Gulf Coast Regional Blood Center (Houston, TX) as previously described (46). Following isolation, primary human monocytes were cultured in conditions identical to those described above for THP-1 cells. Infection with *E. chaffeensis* was also conducted as previously described, and cells were harvested at 48 hpi for analysis.

### Statistical analysis

All data are represented as the means ± standard deviation of data obtained from at least three independent biological replicates, unless otherwise stated in figure legends. Statistical tests are indicated in figure legends and were performed using GraphPad Prism 10 software. *P* < 0.05 was considered statistically significant.
